# A Co-diagnosis of Crohn Disease and Autoimmune Diabetes in an Adolescent Patient

**DOI:** 10.1097/PG9.0000000000000265

**Published:** 2022-10-20

**Authors:** Ioanna Chranioti, George Vartzelis, Despoina Maritsi, Maria Tsolia

**Affiliations:** From the * Pediatric Department, General Hospital of Ierapetra, Crete, Greece; † Second Pediatric Department, National and Kapodistrian University of Athens, Medical School, “P. & A. Kyriakou” Children’s Hospital, Athens, Greece.

**Keywords:** inflammatory bowel disease, type 1 diabetes, immune-mediated diseases, ileocolonoscopy

## Abstract

Inflammatory bowel disease (IBD) is a lifelong, immune-mediated disorder that often occurs in childhood and is becoming increasingly common worldwide. Diagnosis of IBD in children remains difficult due to the spectrum of symptoms, including gastrointestinal and extraintestinal manifestations. Type 1 diabetes mellitus (T1D) is one of the most common autoimmune diseases in children and adolescents. Classic manifestations of T1D in young people include polyuria, polydipsia, abdominal pain, weight loss, and ketoacidosis. However, children with autoimmunity of pancreatic β-cells may remain euglycemic and asymptomatic for many years. An accurate and prompt diagnosis of IBD and T1D is particularly important in children because they can negatively affect growth, psychosocial function and overall well-being. We present a case in which a previously healthy child was co-diagnosed with Crohn disease and T1D during a routine pediatric evaluation in the outpatient clinic of a peripheral secondary hospital.

## INTRODUCTION

Although the incidence and prevalence of inflammatory bowel disease (IBD) have increased in recent years, IBD remains an unusual diagnosis among pediatricians in primary and secondary health care. Patients and/or parents may also underestimate symptoms and delay in seeking medical advice. Herein, we review the case of an adolescent patient with Crohn disease (CD) and coexisting asymptomatic type 1 diabetes mellitus (T1D) at the initial diagnosis.

## CASE PRESENTATION

A previously healthy, 12-year-old Caucasian boy presented to our outpatient clinic for a routine pediatric evaluation. A review of his recent medical history revealed intermittent abdominal pain and diarrhea over the last two months and an intended weight loss of approximately 7 kg over the last four months. Emesis, fever, hematochezia, joint pain, and skin changes were excluded. The patient’s family history was unremarkable.

Upon examination, his vital signs were found to be normal. The patient was pale with normal heart sounds and a grade 2 systolic murmur. His abdomen was soft and nontendered with normal bowel sounds and no palpable masses. The rectal examination was normal. His chest, oral mucosa and skin were clear. No palpable lymph nodes were observed. The patient had a body mass index of 15 kg/m² and his sexual maturity rating was of stage 2. The initial laboratory workup was notable for hypochromic microcytic anemia (hemoglobin 9.4 mg/dL, mean corpuscular hemoglobin 20.5 pg, mean corpuscular volume 67.5 fL) with normal ferritin levels of 70 ng/mL. The erythrocyte sedimentation rate was within normal limits, while the C-reactive protein was elevated at 5.7 mg/dL (normal range < 0.5 mg/dL). In addition, increased fasting blood glucose levels of 107 mg/dL and hypoalbuminemia of 3.2 g/dL were noted. The remaining complete blood count, urine with electrolytes, and liver and thyroid function test results were within normal limits. IBD and prediabetes were suspected, so a more extended workup was performed.

The fecal occult blood test results were positive and fecal calprotectin levels were significantly elevated at 530 mg/kg. Multiple stool specimens for microscopy and culture were used to exclude gastrointestinal infections. The patient also showed positive Crohn serology with anti-*Saccharomyces cerevisiae* antibody positive/pANCA negative. Abdominal ultrasonography revealed thickening of the bowel wall, with increased color Doppler signal denoting hyperemia and decreased peristalsis. Mural changes were noted throughout the sigmoid, the transverse and the ascending colon. An increase in the peristalsis of the small intestine was detected with prominent villi in places without imaging of the terminal ileum. The surrounding mesenteric adipose tissue appeared hyperechoic with many enlarged lymph nodes. Magnetic resonance enterography (MRE) was arranged and the patient was referred to a pediatric gastroenterologist for ileocolonoscopy and esophagogastroduodenoscopy.

At the same time, the patient underwent an oral glucose tolerance test revealing increased fasting blood glucose levels of 110 mg/dL but normal blood glucose levels (113 mg/dL) 2 hours after the ingestion of glucose. In addition, fasting and 1-hour postprandial insulin and C-peptide levels were close to the low-normal range. Glycated hemoglobin of 7.1%, performed twice, was suggestive of the diagnosis of diabetes. The urinalysis results were negative for glucose and ketones. Autoimmune or type 1 diabetes was diagnosed based on the detection of islet autoantibodies in the patient’s serum. Anti-insulin antibodies and islet cell antibodies were positive, whereas tyrosine phosphatase antibodies, glutamic acid decarboxylase antibodies, and zinc transporter 8 antibodies were negative. Serological testing for celiac disease and autoimmune thyroiditis was negative. The patient was advised to have daily measurements of pre- and postprandial blood glucose levels and was referred to a pediatric endocrinologist.

**FIGURE 1.**
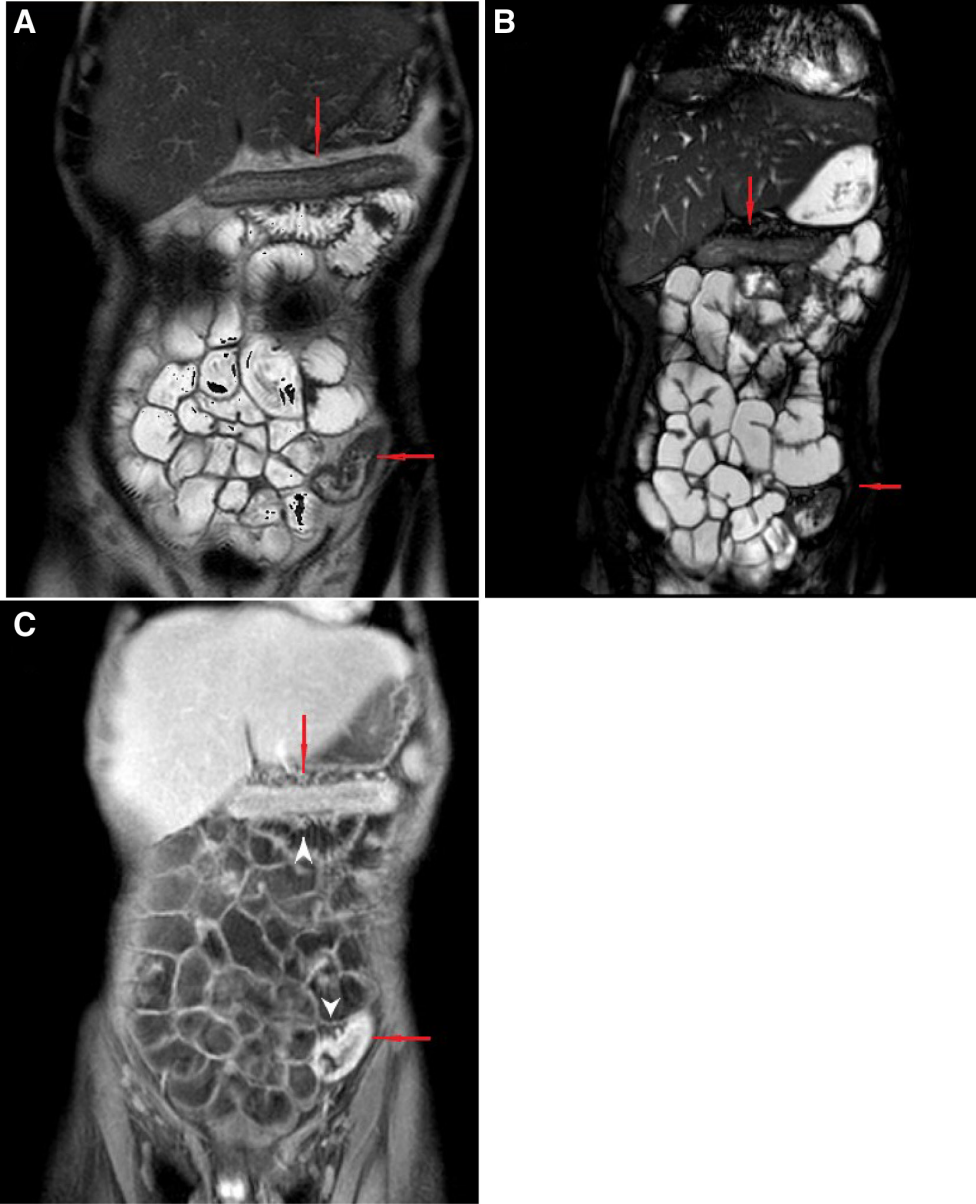
Typical pathological findings of active inflammation in Crohn disease are identified on magnetic resonance enterography with oral polyethylene glycol. A and B) Depiction of wall thickening of the transverse and sigmoid colon (red arrows) on SSFP (A) and SSFSE (B) sequences; (C) Bowel wall enhancement (red arrows) with mesenteric fat thickening and “comb sign” (arrowheads) due to vascular engorgement of mesenteric vessels displayed on gadolinium-enhanced FSPGR sequence. FSPGR = fat-suppressed 3D spoiled gradient echo; SSFP = steady-state free precession; SSFSE = T2-weighted single-shot fast spin-echo.

The patient was evaluated by a pediatric gastroenterologist 3 weeks after his initial presentation at the outpatient clinic, once all the above-mentioned diagnostic evaluations had been completed. However, due to organizational barriers, MRE and endoscopy were performed 1 and 2 months after referral, respectively. MRE demonstrated involvement of the terminal ileum and active inflammatory changes throughout the colon without fistulas, abscesses, or stenoses (Fig. 1). At the time of endoscopy, the patient presented with clinical and laboratory deterioration with hemoglobin 8.8 g/dL, erythrocyte sedimentation rate 67 mm/h, serum albumin 3 g/dL, and fecal calprotectin 1300 mg/kg. The patient had an unintended extra weight loss of 3 kg and an asymptomatic perirectal tag. Esophagogastroduodenoscopy with biopsies was normal. Ileocolonoscopy revealed wall thickening of the terminal ileum with aphthous lesions, and throughout the colon with loss of vascular pattern, friability and ulcerations on visual examination. Those findings were suggestive of CD of moderate endoscopic activity. Histological examination of the ileal and colonic mucosa confirmed the diagnosis of CD. Induction therapy with partial enteral nutrition with a liquid formula and oral prednisolone tapering scheme was commenced. Antitumor necrosis factor (TNF) agent (infliximab) was added to the patient’s treatment 4 weeks later, once his immunization status was updated. Although steroid therapy could worsen hyperglycemia, the pediatric gastroenterologist decided to treat the boy with prednisolone due to the extensive involvement of the whole colon and the unavoidable delay in commencing therapy with anti-TNF agent. At the time of our writing, the patient remains normoglycemic and glycated hemoglobin levels have decreased to 5.9% at week 6 of induction therapy just before the second infusion of infliximab. This improvement in patient’s glycemic status may reflect an effect of anti-TNF agent on glucose metabolism, as it has been already described in young adult patients with T1D and a consequent diagnosis of CD (1). However, long-term follow-up of this patient is needed for a better understanding of the interaction between the two diseases.

## DISCUSSION

This is an unusual case of a co-diagnosis of CD and autoimmune diabetes. Although it has been reported that children with IBD or T1D are more likely to have comorbid immune-mediated diseases (2,3) data on the correlation between T1D and IBD remain conflicting (4–7). The global incidence of pediatric-onset IBD, primarily CD, and T1D has increased over the last decades, with the highest prevalence reported in Europe and North America (8,9). The frequency of ketoacidosis at the onset of T1D has increased in recent years, although its wide geographic variation ranges between 15% and 70% in Europe and North America (10).

Prompt and accurate diagnosis of presymptomatic T1D prevents progression to ketoacidosis, which is linked to poorer long-term glycemic control and sequelae. In addition, timely diagnosis of IBD in children is of great importance, as it reduces complications and can improve prognosis. Days of missed school and prolongation of untreated symptoms affect the physical and psychosocial development of these young patients. Delayed diagnosis is associated with an increased risk of growth failure, delayed puberty, more extensive disease, poorer response to medical treatment, increased need for surgery, and impaired quality of life. Delays may be due to parents and/or patients lacking awareness of relevant signs and symptoms, anxiety about the possibility of receiving bad news, or difficulties in the accessibility of health care facilities. However, medical delays are due to the low suspicion of IBD among general pediatricians, especially those in primary care, as well as the communication and organizational barriers of the health care system.

A detailed history, appropriate clinical examination, and common laboratory tests have the potential to raise clinical suspicion of even rare diagnoses. Although the co-occurrence of CD and T1D is still rare, medical professionals should remember this connection when evaluating patients with suspected IBD or diabetes. In certain cases of asymptomatic patients with β-cell autoimmunity, like our patient, dysglycemia may be triggered by the underlying CD, and treatment and control of the latter may delay the progression of diabetes as well. For instance, increased leakiness of the mucosal barrier for bacterial products or systemic inflammation due to CD may have induced insulin resistance in this patient. Further studies are required for raising awareness about the association between the two diseases.

## References

[1] Timper K Hruz P Beglinger C. Infliximab in the treatment of Crohn disease and type 1 diabetes. Diabetes Care. 2013;36:e90–e91. 23801815 10.2337/dc13-0199 PMC3687309

[2] Kappelman MD Galanko JA Porter CQ. Association of paediatric inflammatory bowel disease with other immune-mediated diseases. Arch Dis Child. 2011;96:1042–1046. 21903597 10.1136/archdischild-2011-300633

[3] Kakleas K Soldatou A Karachaliou F. Associated autoimmune diseases in children and adolescents with type 1 diabetes mellitus (T1DM). Autoimmun Rev. 2015;14:781–797. 26001590 10.1016/j.autrev.2015.05.002

[4] Jasser-Nitsche H Bechtold-Dalla Pozza S Binder E. Comorbidity of inflammatory bowel disease in children and adolescents with type 1 diabetes. Acta Paediatr. 2021;110:1353–1358. 33119925 10.1111/apa.15643 PMC7984099

[5] Virta LJ Kolho KL. The risk of contracting pediatric inflammatory bowel disease in children with celiac disease, epilepsy, juvenile arthritis and type 1 diabetes–a nationwide study. J Crohns Colitis. 2013;7:53–57. 22445838 10.1016/j.crohns.2012.02.021

[6] Bar Yehuda S Axlerod R Toker O. The association of inflammatory bowel diseases with autoimmune disorders: a report from the epi-IIRN. J Crohns Colitis. 2019;13:324–329. 30304371 10.1093/ecco-jcc/jjy166

[7] Lu S Gong J Tan Y. Epidemiologic association between inflammatory bowel diseases and type 1 diabetes mellitus: a meta-analysis. J Gastrointestin Liver Dis. 2020;29:407–413. 32919423 10.15403/jgld-798

[8] Mayer-Davis EJ Kahkoska AR Jefferies C. ISPAD clinical practice consensus guidelines 2018: definition, epidemiology, and classification of diabetes in children and adolescents. Pediatr Diabetes. 2018;19 Suppl 27:7–19. 10.1111/pedi.12773 PMC7521365 30226024

[9] Benchimol EI Fortinsky KJ Gozdyra P. Epidemiology of pediatric inflammatory bowel disease: a systematic review of international trends. Inflamm Bowel Dis. 2011;17:423–439. 20564651 10.1002/ibd.21349

[10] Wolfsdorf JI Allgrove J Craig ME; International Society for Pediatric and Adolescent Diabetes. ISPAD clinical practice consensus guidelines 2014. Diabetic ketoacidosis and hyperglycemic hyperosmolar state. Pediatr Diabetes. 2014;15 Suppl 20:154–179. 25041509 10.1111/pedi.12165

